# Autoimmune Hepatitis-Primary Biliary Cholangitis Overlap in a Case of Pulmonary Arterial Hypertension and Sjögren's Syndrome: Occam's Razor or Hickam's Dictum?

**DOI:** 10.7759/cureus.81730

**Published:** 2025-04-04

**Authors:** Simi Tahiliani

**Affiliations:** 1 Internal Medicine, Atal Bihari Vajpayee Institute of Medical Sciences (ABVIMS) and Dr. Ram Manohar Lohia Hospital, New Delhi, IND

**Keywords:** autoimmune hepatitis, overlap syndrome, paris criteria, primary biliary cholangitis, pulmonary hypertension, sjögren's syndrome

## Abstract

Right-sided heart failure can lead to abnormalities in liver function tests due to hepatic congestion, a condition known as congestive hepatitis. This condition typically responds to diuretics, as well as salt and fluid restriction. However, it is crucial to consider other potential causes of liver function abnormalities if they persist despite optimal therapeutic interventions, to avoid missing treatable conditions.

I present the case of a 37-year-old woman with right heart failure secondary to pulmonary hypertension and Sjögren's syndrome. Initially, her liver function test abnormalities were attributed to congestive hepatitis. However, during follow-up, it was noted that despite the resolution of her ascites, pleural effusion, and pedal edema, her liver function tests remained abnormal. This persistence prompted an investigation for an alternative cause.

Subsequent testing revealed the presence of anti-nuclear antibodies, anti-mitochondrial antibody (AMA) positivity, and speckled protein 100 (Sp100) positivity, as well as elevated immunoglobulin G (IgG) levels. Additionally, a liver biopsy demonstrated interface hepatitis, lymphoplasmacytic infiltrates, and the destruction of medium-sized bile ducts. These findings were consistent with an overlap of autoimmune hepatitis and primary biliary cholangitis.

She was started on prednisolone, azathioprine, and ursodeoxycholic acid. On follow-up, her liver function tests gradually returned to baseline, and her functional status improved.

## Introduction

The 6th World Symposium on Pulmonary Hypertension (WSPH) established a new diagnostic threshold for pulmonary hypertension, defining it as a mean pulmonary arterial pressure greater than 20 mmHg [1]. Pulmonary vascular resistance is considered elevated when it exceeds 2 Wood units. In pre-capillary hypertension, the pulmonary capillary wedge pressure (PCWP) is ≤15 mmHg, whereas in post-capillary hypertension, it is >15 mmHg, with pulmonary vascular resistance remaining below 2 Wood units [2]. The definitive diagnosis is made through right heart catheterization, which also allows for vasoreactivity testing.

Diagnostic evaluation includes a thorough drug history, particularly for fenfluramine, L-tryptophan, amphetamines, and toxic rapeseed oil. Secondary causes such as connective tissue diseases (e.g., scleroderma), schistosomiasis, HIV infection, and porto-pulmonary hypertension must be ruled out. In unexplained cases, transesophageal echocardiography should be performed to detect an underlying atrial septal defect, which may be missed on transthoracic echocardiography. Additionally, genetic mutations such as bone morphogenetic protein receptor type 2 (BMPR2) are associated with a poorer prognosis.

The management of pulmonary arterial hypertension (PAH) is guided by vasoreactivity testing, while its prognosis is assessed using the six-minute walk test. A positive vasoreactivity test suggests potential benefit from dihydropyridine calcium channel blockers. Patients with a negative vasoreactivity test are managed with endothelin receptor antagonists, prostacyclin analogs, phosphodiesterase inhibitors, guanylate cyclase stimulators, and selexipag.

Connective tissue disease-associated pulmonary hypertension is the second most common cause after idiopathic PAH [3]. While both limited and diffuse systemic sclerosis are well-known contributors, the association of isolated Sjögren's syndrome with WHO Group 1 pulmonary hypertension is rare. Although isolated case reports exist [4], no formal study has established the prevalence of pulmonary hypertension in Sjögren's syndrome. However, given that Sjögren's syndrome primarily involves autoimmune epithelitis, it is plausible that pulmonary artery involvement could lead to intimal thickening and muscular hypertrophy.

Sjögren's syndrome has a strong association with primary biliary cholangitis (PBC) and, less commonly, autoimmune hepatitis (AIH) [5]. The Paris Criteria aid in diagnosing overlap syndrome, requiring the presence of at least two of the following three criteria for PBC [6]: (1) anti-mitochondrial M2 antibody (AMA-M2) positivity, (2) elevated alkaline phosphatase (ALP) >3 times the upper limit or gamma-glutamyl transferase (GGT) >5 times the upper limit, and (3) florid duct lesions on liver histopathology.

The diagnosis of AIH as per the simplified criteria requires a score of at least 6 out of 8 for a "probable" diagnosis and 7 or above for a "definitive" diagnosis, as per the simplified criteria for AIH: (1) hepatitis B and C serologies negative by the enzyme-linked immunosorbent assay (ELISA) technique, (2) immunoglobulin G (IgG) level >1.1 the upper limit of normal, (3) anti-nuclear antibody (ANA) titre >1:80 or anti-smooth muscle antibody (ASMA)/liver kidney microsomal type 1 (LKM1) >1:40, and (4) presence of hepatocyte rosettes, interface hepatitis, lymphoplasmacytic infiltrates, or emperipolesis on liver histopathology.

## Case presentation

A 37-year-old woman with no known comorbidities, residing in Bihar, India, presented with exertional shortness of breath for two months. The onset was insidious and gradually progressed from the modified Medical Research Council (mMRC) grade 2 to grade 4. The symptoms were associated with bilateral lower limb swelling, abdominal distension, and yellow discoloration of the eyes. There was no history of chest pain, syncope, or palpitations. She did not report hemoptysis, cough with expectoration, orthopnea, decreased urine output, or frothy urine. Additionally, there was no history of fever, night sweats, or weight loss. She experienced a loss of appetite and early satiety but had no history of joint pain, dry mouth, dry eyes, recurrent dental infections, oral ulcers, hair loss, photosensitive skin rash, recurrent abortions, skin tightening over the face and hands, or blue discoloration of digits upon cold exposure.

On examination, the patient was thinly built, with a body mass index (BMI) of 11.78 kg/m². She appeared pale and icteric, with bilateral pitting pedal edema. Her jugular venous pressure (JVP) was elevated at 8 cm above the sternal angle, with no specific waveform visible. Her blood pressure was 96/70 mmHg in the right arm while sitting, with a pulse rate of 120/min, regular in rhythm, low in volume, and rapid. Oxygen saturation on room air was 96%. Chest auscultation revealed a grade IV/VI pansystolic murmur at the lower left sternal border, which increased with inspiration, suggestive of tricuspid regurgitation. Breath sounds were reduced bilaterally in the infrascapular and infra-axillary regions. An abdominal examination revealed shifting dullness. The liver was palpable 4 cm below the right costal margin in the mid-clavicular line, firm, and non-tender.

Blood investigations revealed a mixed pattern of jaundice, while the renal function tests and serum electrolytes were within normal limits. The relevant findings are summarized in Table 1. A 12-lead electrocardiogram (ECG) showed sinus tachycardia, while a chest X-ray (posteroanterior (PA) view) revealed cardiomegaly with straightening of the left heart border.

**Table 1:** Blood investigations on admission with reference range AST: aspartate aminotransferase; ALT: alanine aminotransferase; ALP: alkaline phosphatase; GGT: gamma-glutamyl transferase

Parameter and unit	Patient value	Reference range
Hemoglobin (g/dL)	10.1	12-16
Total leucocyte count (/mm^3^)	10,400	4000-11,000
Platelet count (lakhs)	1.64	1.5-4.0
Total bilirubin (mg/dL)	3.5	0.2-1.2
Direct bilirubin (mg/dL)	2	0-0.3
AST (IU/L)	394	9-32
ALT (IU/L)	255	8-40
ALP (IU/L)	524	20-140
GGT (IU/L)	190	8-40

**Figure 1:**
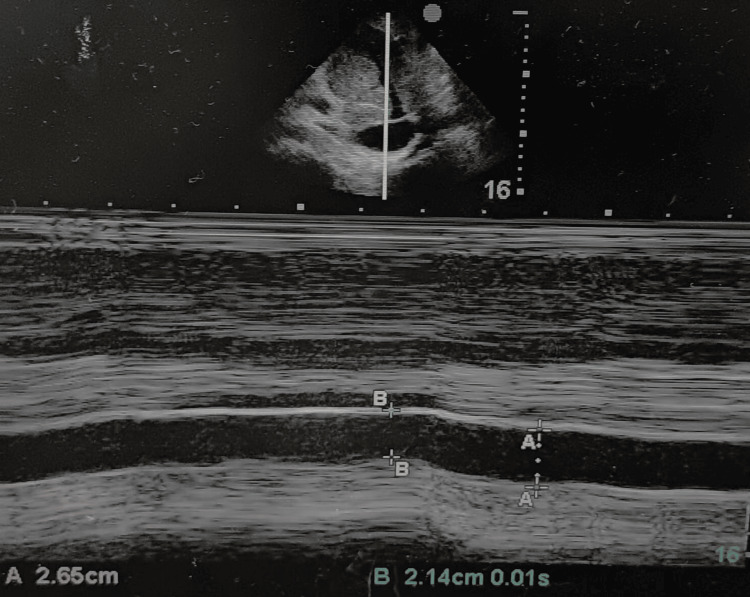
Point-of-care ultrasound showing a dilated inferior vena cava of 2.65 cm diameter with <50% collapsibility

An abdominal ultrasound demonstrated an enlarged inferior vena cava (IVC) with a diameter of 2.65 cm and <50% collapsibility (Figure 1), along with hepatosplenomegaly, moderate ascites, and bilateral pleural effusion. Notably, the portal vein diameter was normal, ruling out porto-pulmonary hypertension.

**Figure 2:**
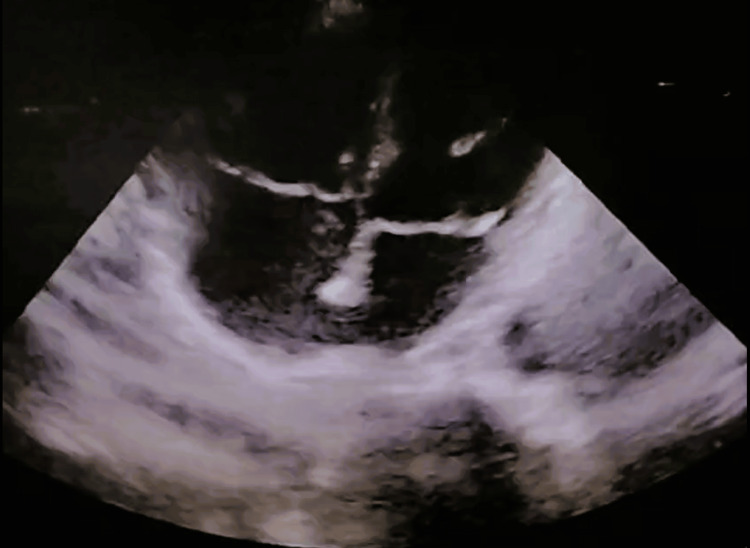
Apical four-chamber view showing a dilated right atrium and right ventricle

A transthoracic 2D echocardiogram showed a dilated right atrium and right ventricle (Figure 2), a D-shaped left ventricle, and torrential tricuspid regurgitation. The tricuspid regurgitant jet velocity was measured at 3.6 m/s, with a right ventricular systolic pressure (RVSP) of 70 mmHg. The left ventricular ejection fraction was 55-60%.

**Figure 3:**
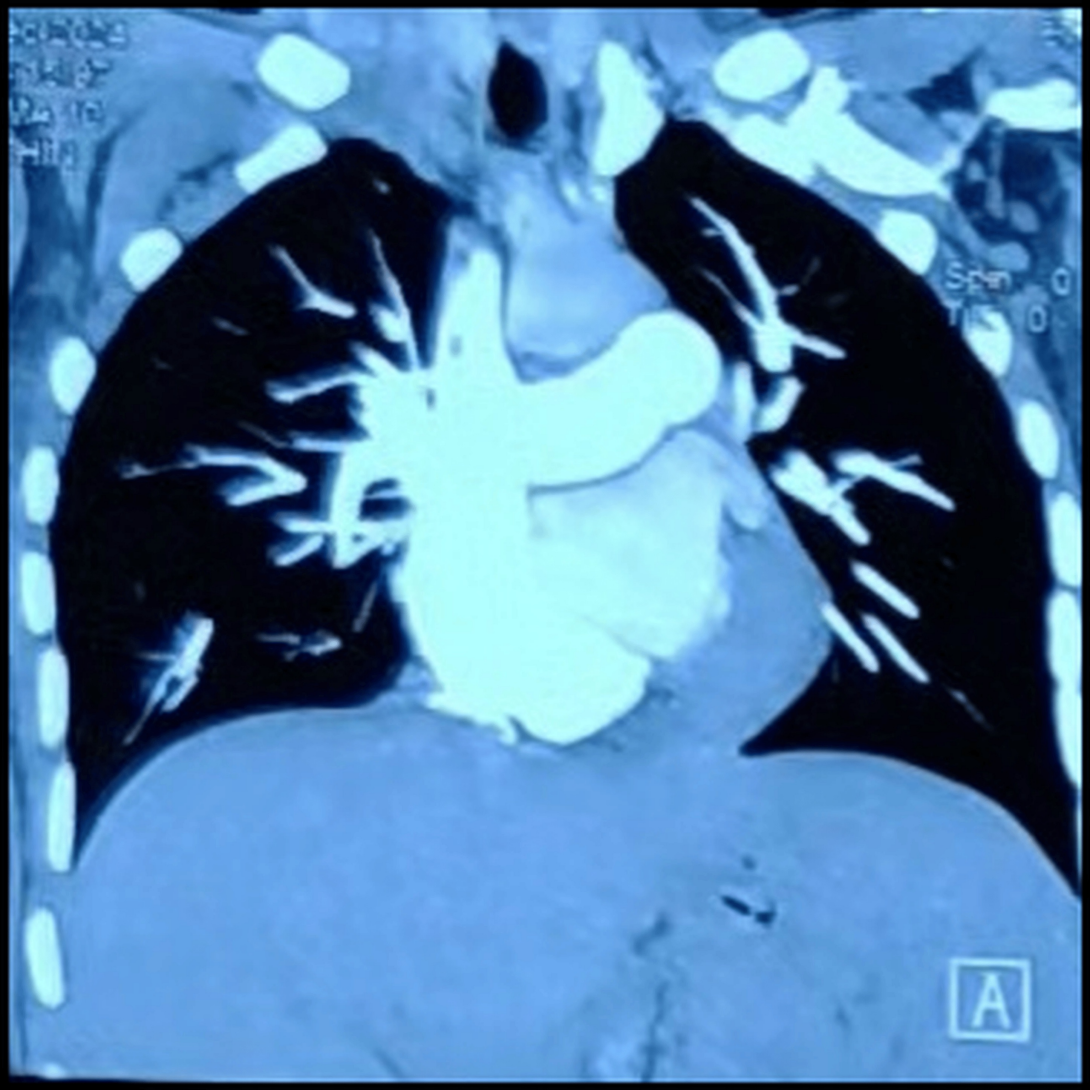
Contrast CT scan of the chest (coronal section) showing an enlarged main pulmonary artery, with normal lung parenchyma

Based on these findings, the patient was diagnosed with right heart failure, congestive hepatitis, and iron deficiency anemia. Management included salt and fluid restriction along with intravenous diuretics. The tricuspid regurgitation was attributed to underlying pulmonary hypertension, prompting a contrast-enhanced CT scan of the chest with pulmonary angiography. The scan revealed an enlarged pulmonary artery, with a pulmonary artery-to-aorta ratio of >1, along with right atrial and right ventricular enlargement (Figure 3). However, the pulmonary parenchyma was normal, and no thrombi were detected.

**Figure 4:**
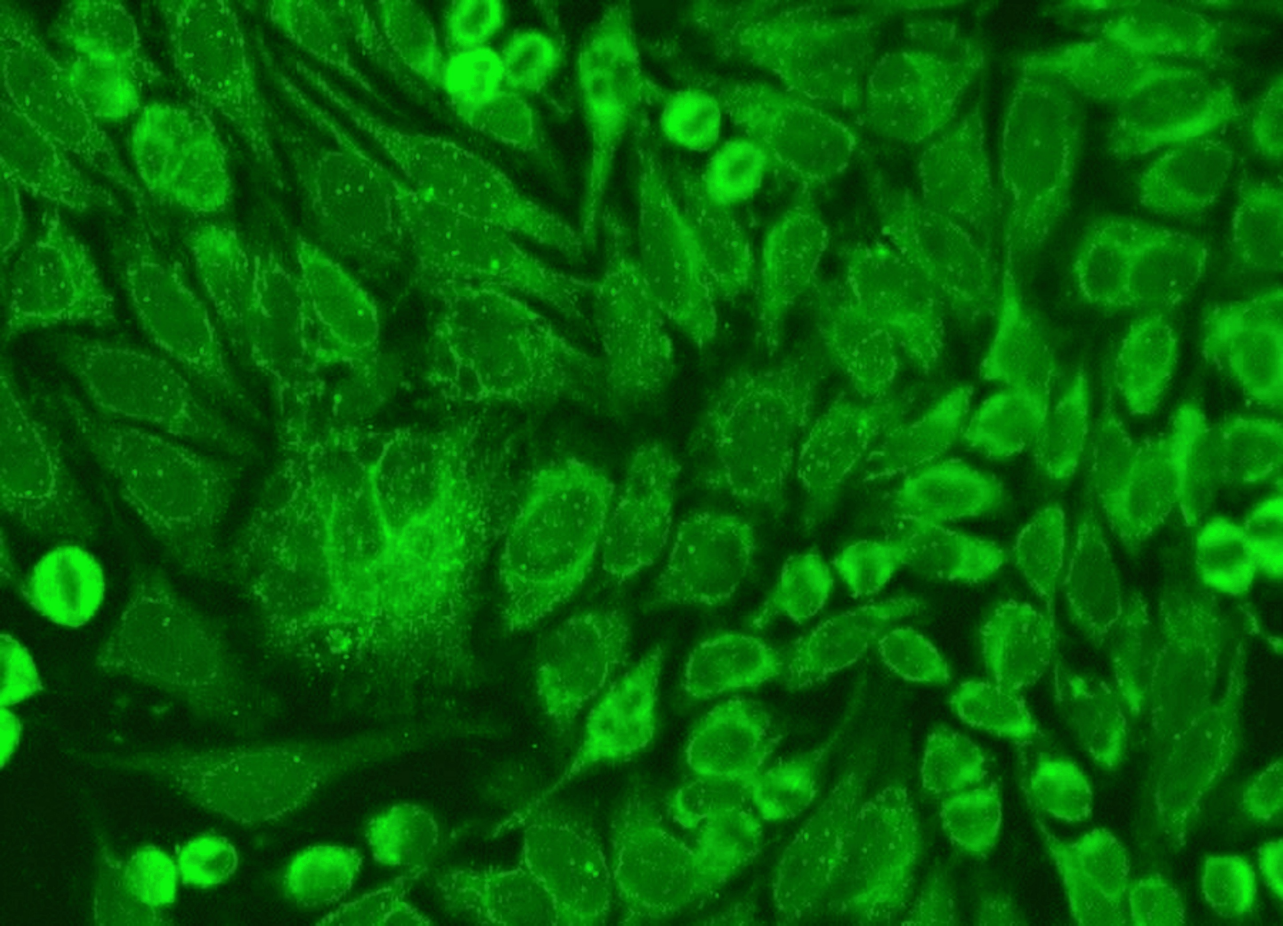
ANA by immunofluorescence showing cytoplasmic pattern, 3+ intensity ANA: anti-nuclear antibody

A detailed drug history was taken to rule out drug- or toxin-induced PAH. Retroviral serology was negative. A transesophageal echocardiogram ruled out an atrial septal defect. ANA testing via immunofluorescence was positive at a 1:100 titre with 3+ intensity and a cytoplasmic pattern (Figure 4). The extractable nuclear antigen (ENA) profile was strongly positive for SS-A antibodies. However, the Schirmer test and ocular staining were negative. A labial biopsy revealed lymphocytic sialadenitis in the minor salivary glands, with a focus score of 2. The vasoreactivity test was negative. Her REVEAL Registry 2.0 score for PAH was calculated to be 8, suggestive of intermediate risk. The patient was initiated on ambrisentan, riociguat, and sildenafil for PAH.

On follow-up, her pleural effusion, ascites, and pedal edema were resolved with diuretics and salt/water restriction. However, her liver function tests remained persistently elevated, prompting further investigation beyond congestive hepatitis. Serology for hepatitis B and C was negative. The AIH profile showed AMA-M2 and speckled protein 100 (Sp100) positivity. Additionally, IgG levels were significantly elevated at 2664 IU/L (normal <1445 IU/L). Based on AMA-M2 positivity, ALP >3 times the upper limit, ANA positivity, IgG elevation >1.1 times the upper limit, and aspartate aminotransferase/alanine aminotransferase (AST/ALT) >5 times the upper limit of normal, the patient met the Paris Criteria for AIH-PBC overlap syndrome.

**Figure 5:**
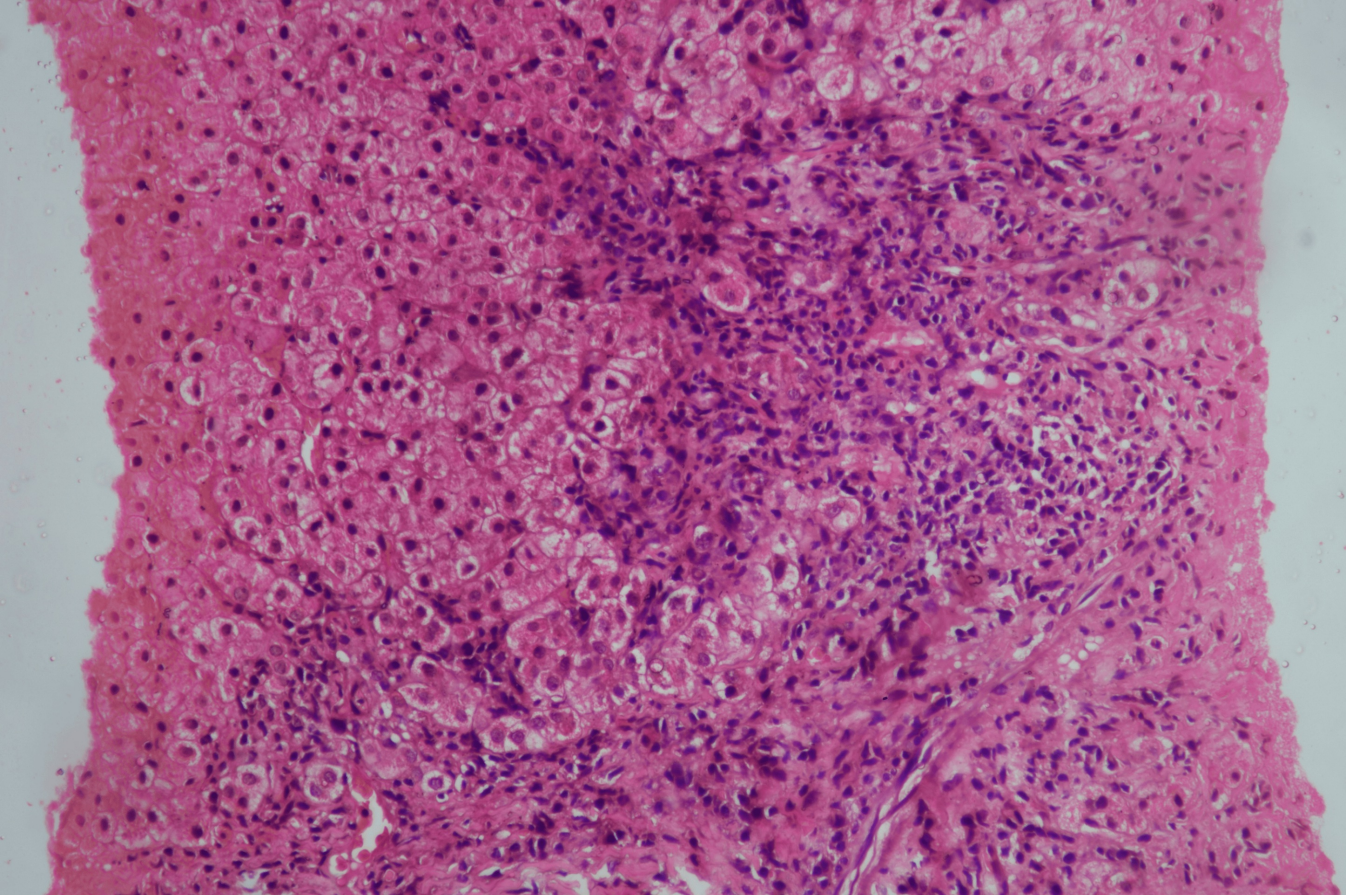
Liver histopathology, 20× magnification, hematoxylin and eosin stain, showing interface hepatitis

**Figure 6:**
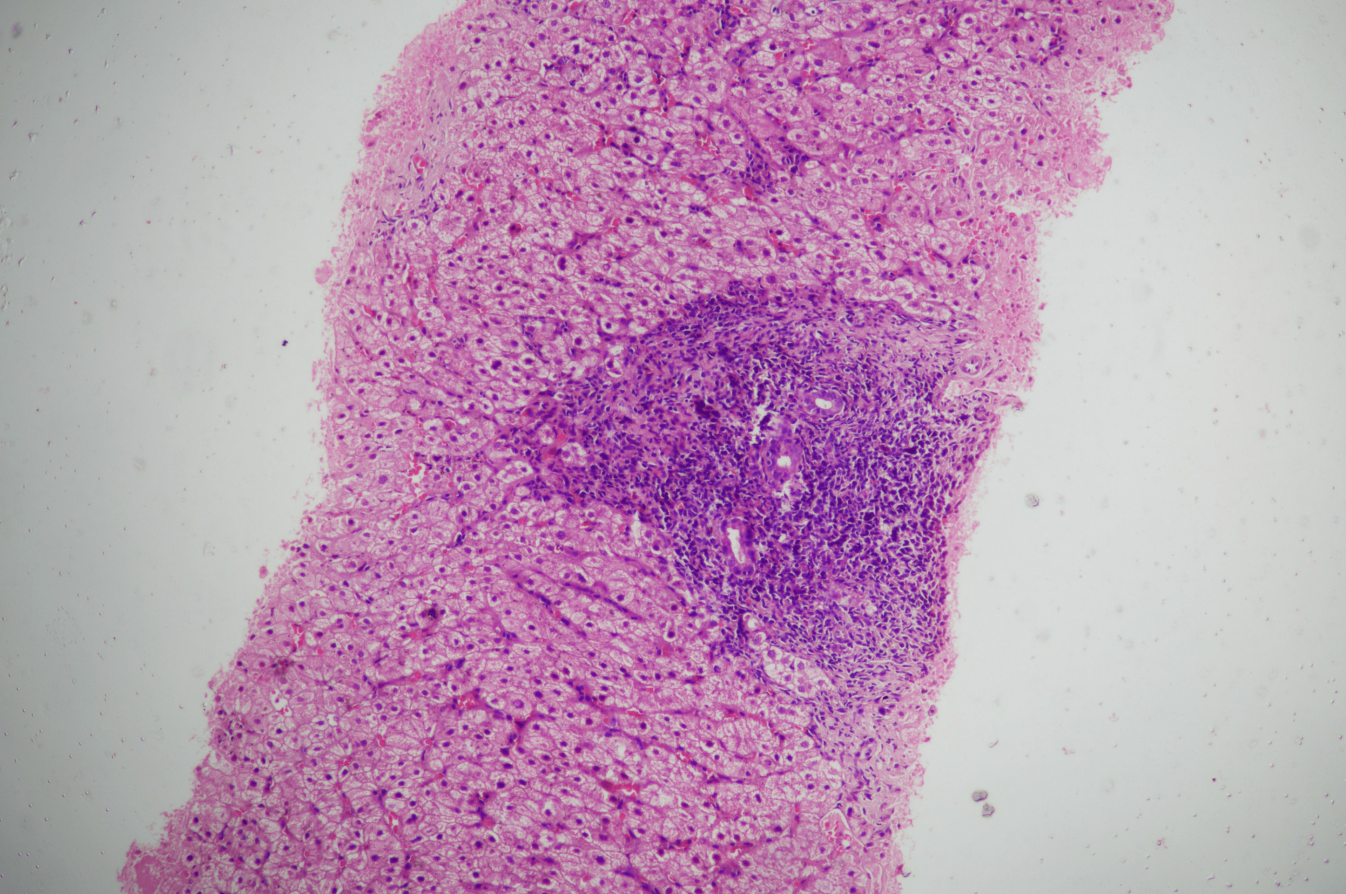
Liver histopathology, 20× magnification, hematoxylin and eosin stain, showing lymphoplasmacytic infiltrates

**Figure 7:**
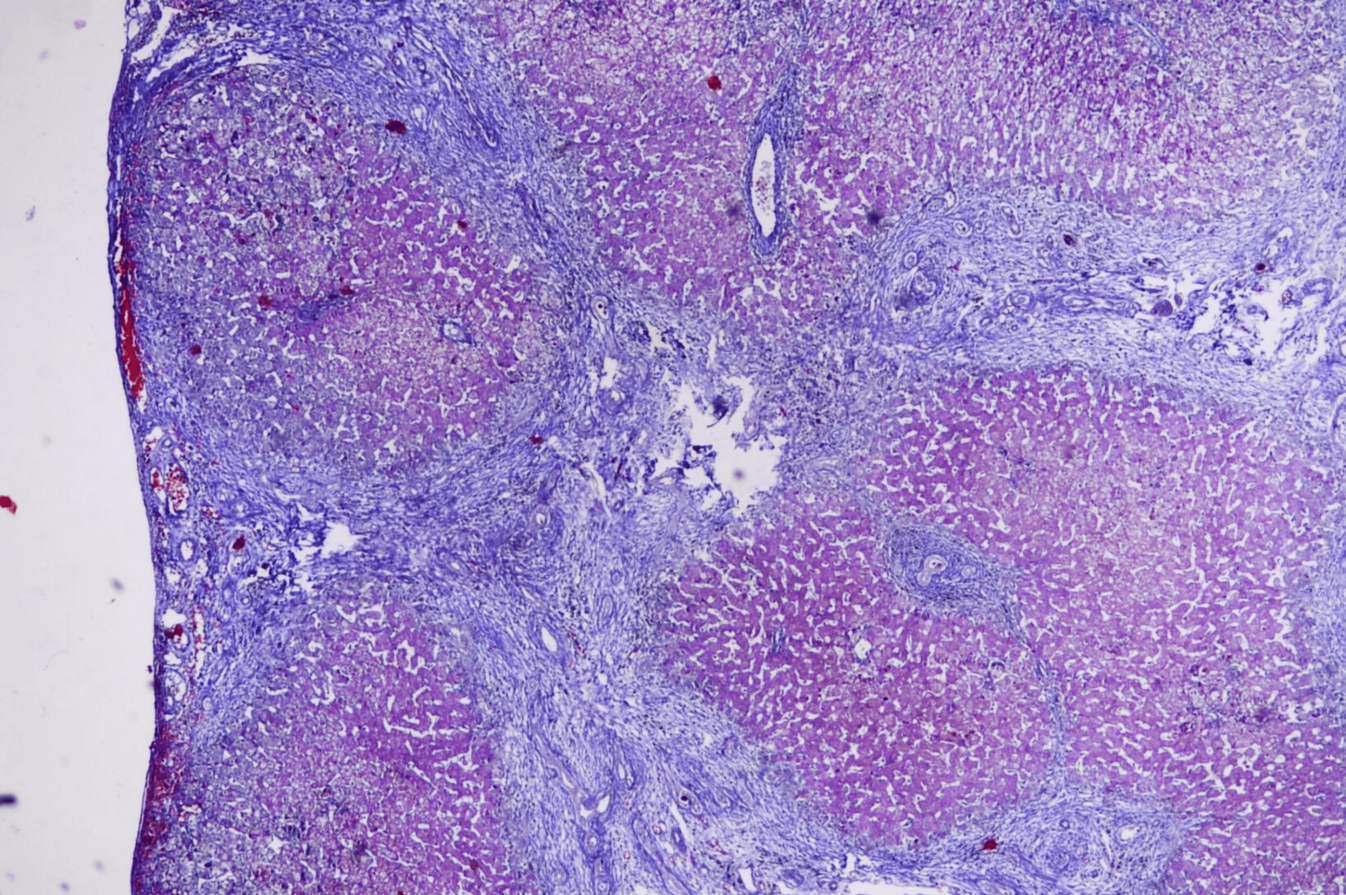
Liver histopathology, Masson's trichrome stain, showing bridging fibrosis

An ultrasound-guided liver biopsy was performed, yielding a 1.2 cm core of liver tissue. Staining with hematoxylin and eosin and Masson's trichrome demonstrated interface hepatitis (Figure 5), lymphoplasmacytic infiltrates (Figure 6) with bridging fibrosis (Figure 7), and medium-sized bile duct destruction, consistent with AIH-PBC overlap syndrome.

The patient was started on ursodeoxycholic acid and prednisolone at 1 mg/kg, with the serial monitoring of liver function tests. Gradually, azathioprine was introduced, and prednisolone was tapered to 5 mg once daily over the course of six months. Over time, liver enzymes and total bilirubin levels decreased, and the patient showed symptomatic improvement. She remained under close follow-up for therapy-related side effects.

## Discussion

Four diseases existed concomitantly in our patient. The presence of pulmonary hypertension led to progressive right atrial and right ventricular dilatation. This resulted in functional tricuspid regurgitation due to annular dilatation. As a result of increasing back pressure, this led to the appearance of ascites, pedal edema, and pleural effusion. At the same time, she also had Sjögren's syndrome and PBC, along with AIH, which led to a persistently deranged liver function test.

Connective tissue disorders commonly linked to PAH include systemic sclerosis, mixed connective tissue disorder, and systemic lupus erythematosus. Although it is plausible for primary Sjögren's syndrome to cause PAH, there are fewer studies available to cite the exact incidence. A study by Kobak et al. from Turkey [7] found the incidence to be 23.4% by the Doppler echocardiography method. Another study by Coppi et al. from Italy [8] found the prevalence of PAH in primary Sjögren's syndrome to be 1.6%. Loss of self-tolerance can incite an immune-mediated attack against multiple target organs, leading to the coexistence of PAH and other autoimmune diseases.

The mechanism of PAH in pre-existing AIH can be attributed to porto-pulmonary hypertension, a complication of long-standing cirrhosis. However, our patient did not have any features of cirrhosis, and AIH was diagnosed at a later point due to persistent liver function test abnormalities despite the resolution of congestive symptoms. 

The management of connective tissue disease-related PAH requires the addition of immunosuppression to the existing armamentarium of drugs that reduce the pulmonary vascular resistance and vascular remodeling. Thus, disease-modifying drugs like azathioprine played a dual role in our patient, targeting both AIH and connective tissue disease-related PAH.

Of note, our patient's BMI was extremely low for her age and gender. Studies show that although overweight and obesity are more commonly associated with PAH, a lower BMI portends a poorer clinical outcome due to greater pulmonary vascular resistance [9]. Thus, our patient requires close follow-up due to the presence of multiple autoimmune diseases, a higher REVEAL score, and the presence of a lower BMI.

## Conclusions

Connective tissue disease-related pulmonary hypertension requires the careful exclusion of multiple possible autoimmune etiologies. While systemic sclerosis, mixed connective tissue disorder, and systemic lupus erythematosus/anti-phospholipid syndrome should be at the forefront of the list, primary Sjögren's syndrome should also be kept in mind as a possible etiology. The presence of such autoimmune diseases warrants the use of disease-modifying immunosuppressants in addition to drugs that reduce pulmonary vascular pressure. While elevated right-sided pressures can lead to liver function abnormalities due to venous congestion, other possible causes should also be kept in mind, since autoimmune diseases tend to cluster. 

This case highlights the importance of maintaining a broad differential diagnosis. While Occam's razor often guides clinical reasoning, Hickam's dictum serves as a valuable reminder that patients can have multiple coexisting conditions. Keeping both principles in mind helps ensure that treatable causes are not overlooked.
